# Influenza A Virus Detection at the Human–Swine Interface in US Midwest Swine Farms

**DOI:** 10.3390/v16121921

**Published:** 2024-12-15

**Authors:** Daniel C. A. Moraes, Michael A. Zeller, Megan N. Thomas, Tavis K. Anderson, Daniel C. L. Linhares, Amy L. Baker, Gustavo S. Silva, Phillip C. Gauger

**Affiliations:** 1 Department of Veterinary Diagnostic and Production Animal Medicine, Iowa State University, Ames, IA 50011, USA; moraes@iastate.edu (D.C.A.M.); mazeller@iastate.edu (M.A.Z.); mneveau@iastate.edu (M.N.T.); linhares@iastate.edu (D.C.L.L.); gustavos@iastate.edu (G.S.S.); 2 Virus and Prion Research Unit, National Animal Disease Center, USDA-ARS, Ames, IA 50011, USA; tavis.anderson@usda.gov (T.K.A.); amy.l.baker@usda.gov (A.L.B.)

**Keywords:** active surveillance, swine, influenza A virus, farm employee, prewean piglets, nursery pigs

## Abstract

This study evaluated influenza A virus (IAV) detection and genetic diversity over time, specifically at the human–swine interface in breeding and nursery farms. Active surveillance was performed monthly in five swine farms in the Midwest United States targeting the employees, the prewean piglets at sow farms, and the same cohort of piglets in downstream nurseries. In addition, information was collected at enrollment for each employee and farm to assess production management practices, IAV vaccination status, diagnostic procedures, and biosecurity. Farm employee and swine samples were screened by IAV reverse transcription real-time polymerase chain reaction (RT-rtPCR), followed by IAV subtyping RT-rtPCR and whole genome sequencing on PCR-positive samples. This study showed higher positivity of IAV RNA detection in nursery pigs compared to prewean pigs, and more whole genome sequences were also obtained in the nursery phase. Surveillance of farm employees revealed two detections of H3N2 representing the 2022–2023 human IAV season, confirming the presence of influenza in farm employees while present at work, and thus highlighting the importance of biosecurity measures at the human–swine interface. This study highlights the importance of routine active surveillance to understand the dynamics of IAV at the farm level in both farm employees and swine.

## 1. Introduction

Influenza A virus (IAV) causes significant economic losses for pig producers primarily due to reduced weight gain [1]. These losses can be more severe in cases where co-infections with other pathogens occur [2,3]. In addition, IAV represents a potential and continuous threat to public health [3,4]. IAV is from the family *Orthomyxoviridae* and has a genome consisting of linear negative-sense, single-stranded RNA divided into eight segments. The virus is enveloped, and its surface is characterized by glycoproteins that include the hemagglutinin (HA) and neuraminidase (NA). The envelope surrounds eight helically symmetrical nucleocapsid segments of different sizes: PB2 (Polymerase Basic 2), PB1 (Polymerase Basic 1), PA (Polymerase Acid), HA (Hemagglutinin), NP (Nucleoprotein), NA (Neuraminidase), M (Matrix), and NS (Non-Structural protein) [5,6].

The natural host of IAV is wild waterfowl, but the virus also infects a wide range of mammals, including swine and humans [7]. IAV in swine may cause zoonotic infections and has contributed to previous human influenza pandemics [8]. Most notable is the 2009 H1N1 pandemic (H1N1pdm09), a reassorted IAV with genetic components derived from avian-, swine-, and human-origin IAV [8,9]. The host range of IAV is determined by the specificity of their interactions with sialic acid (SA) receptors, and specific amino acid substitutions at the receptor binding site of the HA can alter receptor-binding specificity and have been associated with interspecies transmission [10]. Swine exhibit a distribution of α2,3-SA and α2,6-SA receptors in their respiratory tracts comparable to humans [11]. Consequently, swine may be infected with avian- and human-origin IAV, and there may be reassortment with swine populations to generate new genotypes of viruses with unique phenotypes.

In addition to pigs harboring unique influenza viruses with zoonotic potential, workers on swine farms also play a critical role in the transmission dynamics between humans and pigs [12]. A study performed in 2006 found that occupational exposure significantly increased a farm employee’s risk of IAV infection from swine and suggested compelling evidence that IAV exposure from swine frequently occurs among workers employed at swine farms [13]. It is, therefore, prudent to consider swine farm employees for sentinel influenza surveillance and routine human-seasonal influenza vaccination for protection [14]. Recently, a study demonstrated approximately 370 independent human-to-swine spillovers of H1N1 and 17 zoonotic infections after 2009, with the frequency of interspecies transmission increasing when the burden of IAV was highest in the human population [15]. These studies suggest that IAV from humans also poses a risk of infection to swine, a phenomenon known as reverse zoonosis.

Another study conducted in Midwestern pig farms in the United States (US) assessed the risk of transmission between pigs and farm workers [12]. In this study, there was evidence of H1N1pdm09 (1A 3.3.2) infection in a farm employee when reporting to work and exposure of several workers to a swine-origin IAV (H1 1A 1.1.3) circulating in the pigs on the farm where they were employed [12]. This highlights the complex bidirectional transmission of IAV that may occur between pigs and farm employees.

Understanding the dynamics of influenza viruses at the human–swine interface is key for designing optimal surveillance and control strategies [16]. Farm employees may have higher exposure to IAV from swine compared to the general public, considering pigs in commercial swine production are often reared in enclosed facilities with high animal density [17]. Consequently, the shared environment between farm employees and swine should be closely examined at the farm level. This study assessed IAV RNA detection and diversity over time at the human–swine interface by testing pigs and farm employees in breeding farms and their respective nurseries.

## 2. Materials and Methods

### 2.1. Overview of Study Design

A prospective longitudinal study investigated IAV RNA detection and genetic diversity in swine populations and farm employees. The eligibility criteria of farms selected for sampling were farms that agreed to sample the pigs in sow farms and linked nurseries and enrollment of at least 2 farm employees. Active surveillance, a process of routine sample collection regardless of clinical signs, was performed on 10 sites (5 breeding herds and 5 downstream nurseries from matched production systems) targeting employees, prewean piglets 2–3 weeks of age at breeding herds, and the same piglets at 6–9 weeks of age in downstream nurseries in the Midwestern US between April 2022 and July 2023.

### 2.2. Ethical Statement

This study was approved by the Institutional Animal Care and Use Committee (IACUC) of Iowa State University (ISU) under protocol number IACUC-21-146. In addition, human subject research was approved through the ISU Institutional Review Board (IRB) protocol number IRB-21-184 and the Institutional Biosafety Committee (IBC) protocol number IBC-21-058.

### 2.3. Sampling Protocol for Piglets and Farm Employees

The sampling protocol for farm employees, prewean, and nursery piglets is summarized in Table 1. The sampling kits were prepared in a research laboratory at the Iowa State University Veterinary Diagnostic Laboratory (ISU VDL). The udder and nasal wipe kits contained a Whirl-Pak bag (Nasco, Fort Atkinson, WI, USA) with 10 mL of DMEM (Gibco Lab Inc., Grand Island, NY, USA) and a 4 × 4-inch sterile cotton gauze pad (Fisher HealthCare, Houston, TX, USA). All kits were prepared in a Biological Safety Cabinet Class II A2 research laboratory at the ISU VDL.

When received, the samples were tested monthly for IAV RNA by RT-rtPCR in the research laboratory. Following the approved IRB protocol, at least two farm employees were voluntarily enrolled for each farm. The farm employee sample was self-collected, anonymized, and only identified by age, ensuring confidentiality and following IRB guidelines. 

The udder wipe sampling procedure followed a previously described methodology [18] and was performed by farm employees or veterinarians. Briefly, the surface of the udder skin was thoroughly wiped with a gauze moistened with Dulbecco’s Modified Eagle Medium (DMEM, Gibco Lab Inc., Grand Island, NY) in the areas of contact where the piglets would suckle and contact the udder skin. Next, one moistened gauze was collected from each sow, with each gauze placed into a separate Whirl-Pak bag (Filtration Group, Austin, TX, USA) containing 10 mL DMEM media, resulting in a total of 10 gauze samples.

The farm employees or farm veterinarians performed the piglet nasal wipe sampling. The pig snouts were wiped with gauze moistened with DMEM media. Next, one wiped gauze per pig snout was placed into a separate Whirl-Pak bag containing 10 mL DMEM, resulting in 15 gauze samples. These procedures followed the methodology previously described [19]. Each sample collection was conducted with disposable gloves replaced between samples and across the litter to prevent contamination.

Oral fluids were collected by tying a cotton rope where the nursery pigs could interact with the material. After approximately 30 min, the chewed cotton rope was wrung using a clean plastic bag, and the resulting fluid was transferred to a 50 mL conical centrifuge tube (Fisher Scientific, Pittsburgh, PA, USA). The oral fluid sampling procedure followed the previously described methodology [20].

### 2.4. Survey for Assessment of Farm Practices

A farm survey was emailed to the swine producers or their veterinarians. It included 27 questions (Supplementary Material A) that could be answered through an attached Microsoft Excel file in the email. The survey asked about farm practices to assess production management, IAV vaccination protocols, diagnostic procedures, and biosecurity. At the time of farm employee enrollment in this study, each participant responded to demographic questions related to age, gender, influenza vaccination history, and length of time working with swine (in years), as approved by the IRB protocol.

### 2.5. IAV Nucleic Acid Extraction, IAV Screening RT-rtPCR, and IAV Subtyping RT-rtPCR

IAV nucleic acid extraction was performed in all samples using the MagMAX Pathogen RNA/DNA isolation kit and a Kingfisher 96 instrument (Thermo Fisher Scientific, Waltham, MA, USA) using the procedures recommended by the manufacturer. For the lysis step, 100 μL of each sample was combined with 240 μL of the lysis-binding solution. Before extraction, internal positive control RNA (VetMAX Xeno internal positive control RNA; Thermo Fisher Scientific) was introduced into the lysis-binding solution at a concentration of 20,000 copies per reaction. This served the purpose of monitoring PCR amplification and detecting potential inhibition. The extraction process involved 300 μL and 450 μL of wash solutions I and II, respectively, and the nucleic acid was eluted into 90 μL of elution buffer. One positive extraction control and one negative extraction control were also included in the extraction plate.

The IAV screening RT-rtPCR procedure was conducted as recommended by the manufacturer (VetMAX™-Gold SIV Detection kit, Life Technologies, Austin, TX, USA). The total volume of RT-rtPCR master mix contained 12.5 μL of 2x Multiplex RT-PCR Buffer, 2.5 μL of Multiplex RT-PCR Enzyme Mix, 1.0 μL Influenza Virus Primer Probe Mix, and 1.0 μL of Nuclease-free Water, totaling 17 μL. Next, 8 μL of template was added to the master mix with a final reaction volume of 25 μL. The assay was conducted on an ABI-7500 Fast system (Thermo Fisher Scientific), using the 7500 Fast System SDS Software Version 1.5.1 for analysis. The ABI-7500 was set to run with the following cycling conditions: one cycle at 48 °C for 10 min, 1 cycle at 95 °C for 10 min, and 40 cycles at 95 °C for 15 s and 60 °C for 45 s. Each RT-rtPCR plate included two positive amplification controls and two negative amplification controls. Additionally, run data were analyzed using the auto baseline with thresholds of the target and Xeno set according to the kit insert. Samples with cycle threshold (Ct) values < 38 were considered positive.

The subtyping RT-rtPCR assay was conducted as recommended by the manufacturer (VetMAX™-Gold SIV Subtyping Kit, Life Technologies, Austin, TX, USA). The total volume of the RT-rtPCR master mix consisted of 12.5 μL of 2x Multiplex RT-PCR Buffer, 2.5 μL of Multiplex RT-PCR Enzyme Mix, 1.0 μL of Influenza Virus Primer Probe Mix (either HA or neuraminidase [NA]), and 1.0 μL of Nuclease-free Water, resulting in a total volume of 17 μL. This process involved the preparation of duplicate mixes, with one mix designated for HA and another for NA, each arranged in separate rows. Subsequently, 8 μL of extract from each IAV-positive sample was added to the master mix, resulting in a final reaction volume of 25 μL for both HA and NA assays. The assay was conducted on an ABI-7500 Fast system (Thermo Fisher Scientific), using the 7500 Fast System SDS Software Version 1.5.1 for analysis. The ABI-7500 was set to run with the same cycling conditions as the screening RT-rtPCR previously described. Additionally, run data were analyzed using auto baseline with thresholds of the target and Xeno set according to the kit insert. Samples with cycle threshold (Ct) values < 38 were considered positive.

### 2.6. Diagnostic Testing

All samples were shipped to the research laboratory at the ISU VDL, received, and transferred to sterile Falcon 5 mL snap cap tubes (Corning, Glendale, AZ, USA). All samples were tested for IAV RNA by RT-rtPCR following the ISU VDL standard operating procedures. A sample was considered positive when the RT-rtPCR cycle threshold (Ct) value was <38, as recommended by the IAV commercial kit (VetMAX™-Gold SIV Detection kit, Life Technologies, Austin, TX, USA).

### 2.7. Whole-Genome and Sanger Gene Sequencing

An IAV RT-rtPCR positive sample (Ct value < 38) with a cycle threshold (Ct) value lower than 35 from a farm employee, prewean piglets, and nursery pigs was submitted monthly to attempt whole genome sequencing (WGS), as samples with lower Ct values were expected to yield successful sequencing results. Sequencing libraries were constructed using TruSeq (Illumina, Inc., San Diego, CA, USA). Next-generation sequencing was performed on an Illumina, Inc., MiSeq platform by following standard Illumina protocols at the ISU-VDL [21]. If a positive sample was not detected through WGS, the sample was submitted for Sanger sequencing of the HA and NA genes performed according to standard operating procedures of the ISU VDL [21]. Sanger sequencing primers are available upon request. The FASTA sequences were classified using octoFLU for WGS and ISU *FLU*ture for IAV Sanger sequencing analysis [22,23]. Only IAV gene sequences of full length without ambiguous bases were included in the phylogenetic analysis: PB2 (2280 bp), PB1 (2275 bp), PA (2151 bp), HA (1695–1701 bp), NP (1497 bp), NA (1410 bp), M (982 bp), and NS (838 bp).

### 2.8. Phylogenetic Sequence Analysis

The obtained FASTA sequences were imported into Geneious Prime 2024.0.5 (https://www.geneious.com, (accessed on 10 February 2024)) and aligned using MAFFT v7.450 with default settings. The aligned sequences were used to construct maximum-likelihood trees using RAxML 8.2.11 version in Geneious Prime [24]. The phylogenetic trees were exported to FigTree software for tree-building methods. Trees were visualized and annotated using FigTree version 1.4.1 (http://tree.bio.ed.ac.uk/software/figtree/, (accessed on 5 April 2024)). Bootstrap values are indicated at each node. The tree was rooted at the midpoint, and nodes were ordered using the decreasing option. Assigned colors were used to identify the surveillance and reference sequences. GenBank numbers for HA and NA sequences are provided in the Supplementary Material (Supplemental Material Table S1). GenBank accession numbers for the internal gene segment sequences are available upon request. Reference sequences in phylogenetic trees can be accessed using the alpha-numeric numbers included in the taxa.

The H3 HA gene nucleotide homology and antigenic motif [25] were also assessed to compare the H3 sequences detected in the farm to the H3 clade used in the autogenous vaccine. The H3 nucleotide homology was calculated from the multiple sequence alignment in Geneious Prime. In addition, the H3 antigenic motif was obtained using the publicly available Amino Acid Sequence Motif Viewing Tool, accessible online by ISU *FLU*ture [22].

### 2.9. Statistical Analysis

Descriptive statistical analyses were conducted to ascertain the frequency of IAV RNA detection by sample type using the R Studio software version 4.1.1 [26]. Given the overall proportion of positivity by each farm and phase (breeding herd and nursery) during this study, farms were classified as lower positivity (IAV positivity ≤ 10%) and higher positivity (IAV positivity > 10%). Next, the Fisher exact test assessed any association between each management practice and the rate of IAV positive detection. The significance was set at *p* ≤ 0.05 to identify differences in the responses among the lower and higher IAV positive farms.

## 3. Results

### 3.1. Farm Employee Survey Responses and RT-rtPCR Results

Complete responses were received from 48 farm employees who voluntarily enrolled from the five farms in the study. Of the farm employees who were enrolled and responded to the survey, 62.5% (30/48) were male and 37.5% (18/48) were female. In the prior 12 months, 29.2% (14/48) responded that they received an influenza vaccine and 66.7% (32/48) responded that they did not receive the human-seasonal IAV vaccine (Table 2). The majority of respondents had been working with swine for more than 2 years (62.5%).

**Figure 1:**
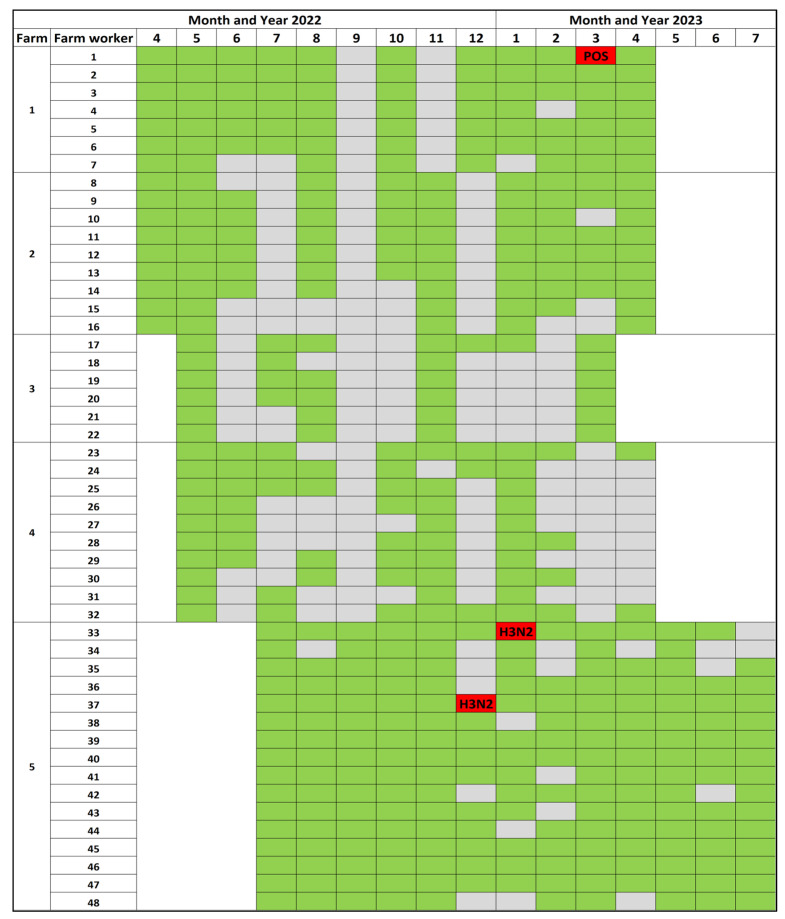
Farm employee IAV RNA RT-rtPCR monthly results by Farms 1–5. Green indicates a negative result, red indicates a positive result, and grey indicates samples were not received. H3N2 represents the subtype of IAV detected. POS represents a RT-rtPCR-positive sample without subtyping information.

The RT-rtPCR results obtained from the farm employees participating in the study between April 2022 and July 2023 had an IAV positivity of 0.67% (3/447), with two positive results recorded within the same farm (Farm 5) with a Ct value of 35.08 (December 2022) and 33.27 (January 2023). Among these two samples, an H3N2 IAV was identified through subtyping RT-rtPCR. In March 2023, a different farm (Farm 1) yielded a positive result; nevertheless, due to its weak Ct value (37.8), the IAV strain could not be identified through IAV RT-rtPCR subtyping and Sanger sequencing methods. Additionally, the farm employee from Farm 1 who had the weak IAV positive detection responded that they received a human-seasonal IAV in the prior 12 months, and the two employees from Farm 5 who were IAV-positive did not receive a human-seasonal IAV vaccine in the prior 12 months. Figure 1 illustrates the samples collected from the farm workers participating in the study and each farm’s monthly IAV screening RT-rtPCR outcomes throughout the study period.

**Figure 2:**
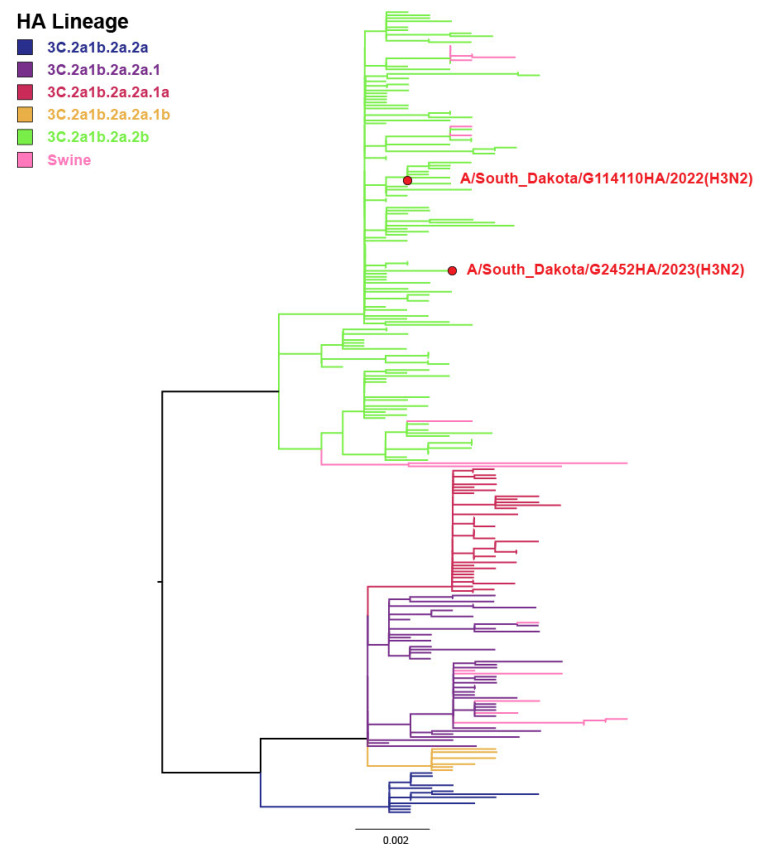
An H3 phylogenetic tree representing two hemagglutinin gene sequences detected from different farm employees during December 2022 and January 2023 from Farm 5 and highlighted in red representing two different H3 strains within the 3C.2a1b.2a.2b clade of human-seasonal IAV.

Two IAV-positive farm employee samples by RT-rtPCR from Farm 5 were sequenced by Sanger methods, and HA sequences were detected for both samples representing the 2022–2023 human-seasonal IAV from the 3C.2a1b.2a.2b clade (Figure 2). The WGS was unsuccessful, likely because of weak RT-rtPCR Ct values. While human-seasonal IAV was occasionally detected among three farm employees, swine-lineage IAV was not detected in human nasal secretions from the farm employees during this study, indicating no evidence of swine-origin IAV transmission to humans during the study period. Thus, these findings demonstrated that farm employees can have detectable human-seasonal IAV in their respiratory tract while at work on a swine farm, although this study did not provide direct evidence of swine-origin IAV transmission to humans or human-seasonal IAV transmission to swine.

### 3.2. Breeding Herd and Nursery Farm Demographics and Management Survey Responses

The breeding herds were distributed among Farm 1, Farm 2, Farm 3, Farm 4, and Farm 5, with 1240, 2700, 5600, 1800, and 5700 sows, respectively. Overall, this surveillance project included 17,040 sows from five breeding herds. Farms 1 and 2 were in Iowa, Farms 3 and 4 were in Illinois, and Farm 5 was in South Dakota. Regarding production management practices in breeding farms, 80% (4/5) reported weaning in a continuous flow, and 80% (4/5) reported weaning piglets weekly. Of the farms, 100% (5/5) responded that they use vaccinated gilts before herd entry using autogenous or farm-specific influenza vaccine products. The use of IAV vaccines varied at other stages of production; while some locations targeted animals as they entered the breeding herd, others used whole-herd vaccination, and the frequency of vaccination also varied (Table 3).

Sixty percent (3/5) of the farms implemented a routine passive influenza surveillance program. When replacing gilts, three of the five farms (60%) reported the gilt source IAV status was negative, and for gilt source influenza antibody status, 60% (3/5) responded that it was unknown. The farm’s specific influenza surveillance protocol goals also varied by farm (Table 4).

For IAV biosecurity practices, 100% (5/5) of the farms recommended employee influenza vaccines. However, 80% (4/5) of the farms responded that farm employees were influenza-vaccinated, and 60% (3/2) recommended using a sick leave policy. For IAV biomanagement practices, 100% (5/5) used nurse sows, and 80% (4/5) said the nurse sows moved between farrowing rooms (Table 4).

For IAV diagnostic testing and surveillance performed in the nursery, 40% (2/5) of the farms reported mixed sources of pigs at the nursery sites, 80% (4/5) of the farms indicated they did not conduct routine IAV surveillance in this population, and 100% (5/5) reported they did not use IAV vaccines in the nursery pigs (Table 5).

Across farms and phases, variations in IAV positivity were observed. Sow farms generally demonstrated lower positivity rates, with Farms 1, 2, 3, and 5 categorized as having lower IAV positivity, with 0%, 2%, 2%, and 1%, respectively, while Farm 4 was classified as having higher IAV positivity, with a 16% detection rate. In contrast, nursery farms consistently showed higher positivity levels, with Farms 1, 2, 4, and 5 with 41%, 16%, 36%, and 37%, respectively, while Farm 3 was classified as having lower IAV positivity, with a 6% detection rate. These findings highlight the increased IAV detection in nursery phases compared to sow farms across the studied sites. Furthermore, no statistical difference (*p* ≥ 0.05) was observed in the assessment of differences in farm management responses between farms with lower and higher IAV positivity (Supplemental Material Table S2).

**Figure 3:**
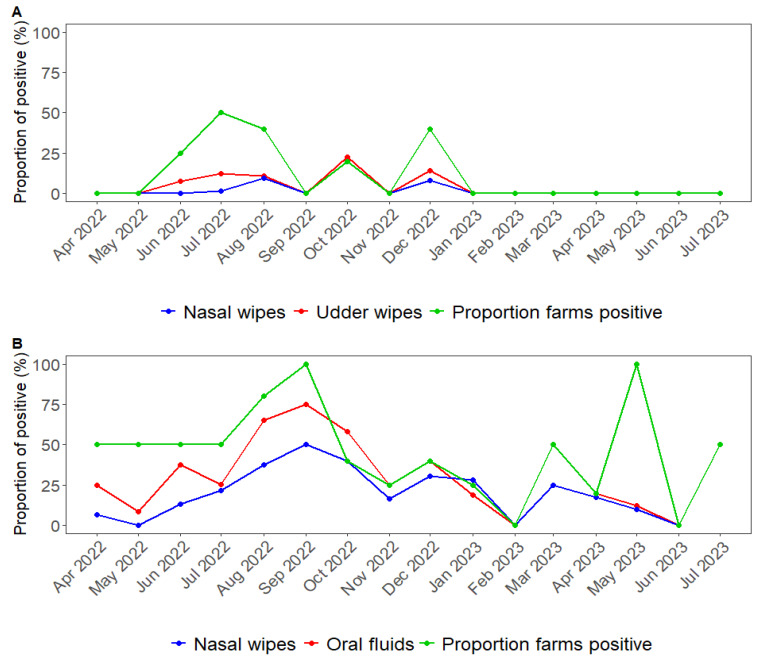
The aggregated proportion of IAV RNA RT-rtPCR positivity per sample type and farm over time. (**A**) Prewean piglets; (**B**) nursery pig phase. The proportion of farms positive (green line) represents the number of farms with at least one sample test positive from the total number of participating farms.

The overall IAV positivity among all sample types for the duration of the study in prewean pigs was 4.76% (57/1198), where nasal wipes had 54.38% (31/57) positive and udder wipes had 45.61% (26/57) positive. In addition, the overall IAV positivity among all sample types during the study in the nursery phase was 29.37% (282/960), where nasal wipes had 72.69% (205/282) positive and oral fluids had 27.31% (77/282) positive (Figure 3). Additionally, the mean Ct value was 34.3 for udder wipes and 34.0 for nasal wipes during the prewean phase. In contrast, during the nursery phase, oral fluid samples had an average Ct value of 30.5, while nasal wipes had a lower average Ct value of 28.7, indicating higher viral loads than the prewean samples.

**Figure 4:**
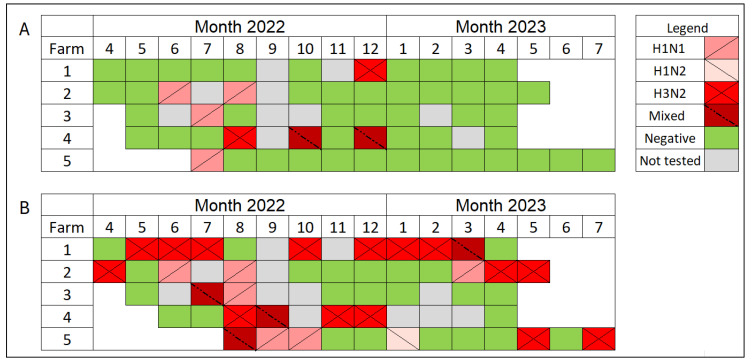
Detection of IAV subtypes by RT-rtPCR by month and farm. (**A**) Prewean phase; (**B**) nursery phase. The legend at the upper right is the IAV subtypes represented by color and stripes: H1N1 (dark pink, a diagonal stripe from bottom left to upper right), H1N2 (light pink, a diagonal stripe from upper left to bottom right), H3N2 (red, two diagonal stripes), Mixed (dark red, a diagonal stripe from upper left to bottom right), Negative (green, no stripes), and Not tested (gray, no stripes).

H1N1 and H3N2 were confirmed by RT-rtPCR in the prewean piglets, with two mixed subtype detections exclusive to Farm 3. The IAV subtypes H1N1, H3N2, and H1N2 were detected in the nursery pigs, with mixed detections identified in all five farms (Figure 4).

**Figure 5:**
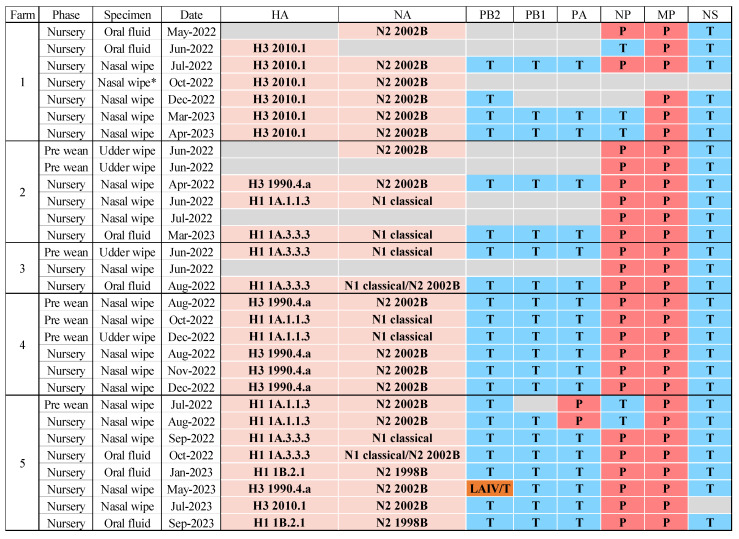
IAV hemagglutinin global classification and neuraminidase US classification. Internal gene constellations represent 6 gene segments from preweaning and nursery pigs in each sequence by farm if sequencing was successful. T (blue) denotes the triple-reassortant internal gene (TRIG) lineage. P (red) represents pandemic lineage. The live attenuated influenza virus (LAIV) vaccine and T (orange) represent mixed vaccine virus and TRIG lineages in the internal gene PB2. Empty cells shaded in grey represent hemagglutinin, neuraminidase, or internal genes that could not successfully sequence. * The nasal wipe sample in the nursery was tested by Sanger sequencing.

The HA sequencing results from samples collected from prewean piglets in breeding herds revealed the presence of various IAV clades (Figure 5). Specifically, the H1 1A.3.3.3 clade was sporadically detected (*n* = 2) in Farm 3. The H1 1A.1.1.3 clade was identified in Farms 4 (*n* = 2) and 5 (*n* = 1) with sporadic detection, and H3 1990.4.a (*n* = 1) was also detected in Farm 4. The N1 classical NA gene was detected in prewean pig samples in Farms 3 (*n* = 1) and 4 (*n* = 2). The N2 2002B NA gene was detected as an endemic infection in Farm 2 (*n* = 1), farm 4 (*n* = 1) and occasionally in Farm 5 (*n* = 1) (Figure 5).

Sequencing results in the nursery pigs revealed HA clades H1 1A.1.1.3 in Farms 2 (*n* = 1) and 5 (*n* = 1) with sporadic detection (Figure 5). Hemagglutinin sequences from the H1 1A.3.3.3 were detected in Farm 2 (*n* = 1), Farm 3 (*n* = 1), and Farm 5 (*n* = 2) with sporadic detection. H1 1B.2.1 was found also in Farm 5 (*n* = 2). H3 2010.1 represented an endemic infection in Farm 1 (*n* = 6) and sporadic detection in Farm 5 (*n* = 1). The H3 1990.4.a clade was found in Farm 2 (*n* = 1) and Farm 5 (*n* = 1) with sporadic detection, and in Farm 4 (*n* = 3) as an endemic infection (Figure 5).

For NA sequencing in samples from nursery pigs, N1 classical swine was detected in Farm 2 (*n* = 2), Farm 3 (*n* = 2), Farm 4 (*n* = 2), and Farm 5 (*n* = 2) with sporadic detection (Figure 5). In addition, NA sequences representing N2 2002B clades were endemically detected in Farm 1 (*n* = 6), Farm 4 (*n* = 3), and Farm 5 (*n* = 4). However, NA 2002B clades were sporadically detected in Farms 2 (*n* = 1) and 3 (*n* = 1). Moreover, clades N2 1998B were detected in Farm 5 in two different months. Furthermore, two mixed NA detections with N1 classical swine and N2 2002B were identified from oral fluid samples from Farms 3 and 5 in the nursery phase (Figure 5).

The six IAV internal genes of PB2, PB1, PA, NP, M, and NS, designated the internal gene constellation, consist of segments originating from the triple reassortant internal gene constellation (TRIG: T) or the 2009 pandemic H1N1 (PDM: P). The internal gene constellation TTTPPT was most often detected in the prewean and nursery phases. In Farm 1, there were three detections of the NP gene representing the TRIG lineage and two NP genes representing the pandemic lineage. Moreover, in the nursery phase, TRIG and live attenuated influenza virus (LAIV) vaccine were detected in PB2 from nasal wipes in Farm 5 (Figure 5).

**Figure 6:**
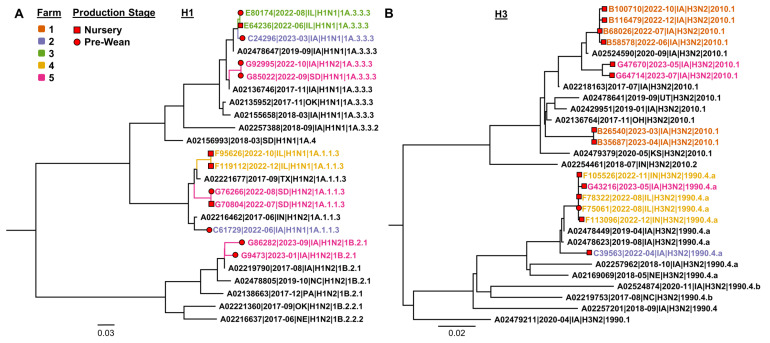
Maximum likelihood influenza A virus hemagglutinin gene phylogenetic trees representing H1 (**A**) and H3 (**B**) subtypes, including sequences generated from the prewean (red circle) and nursery phases (red square). The reference sequences are presented in black. Farm 1 is represented by orange, Farm 2 blue, Farm 3 green, Farm 4 yellow, and Farm 5 pink.

Phylogenetic analysis of HA gene sequences revealed the circulation of H1 1A.3.3.3 in prewean and nursery stages in Farm 3 (represented by green). Also, in both production stages, H1 1A.1.1 sequences were detected in Farm 5 (represented by pink). In Farm 4 (represented by orange), H3 1990.4.a was detected in both production stages (Figure 6).

**Figure 7:**
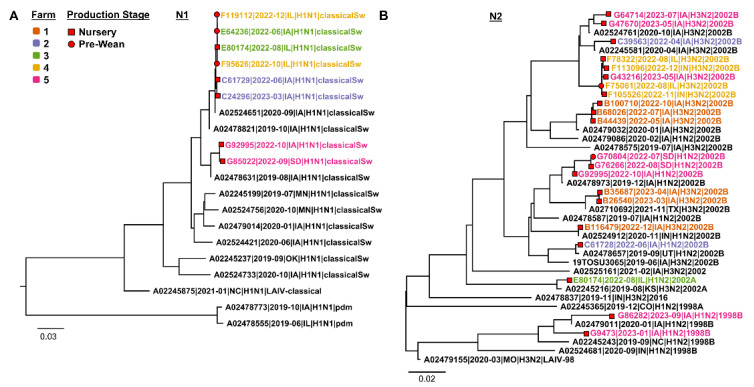
Maximum likelihood influenza A virus neuraminidase gene phylogenetic trees representing N1 (**A**) and N2 (**B**) subtypes, including sequences generated from prewean (red circle) and nursery phases (red square). The reference sequences are presented in black. Farm 1 is represented by orange, Farm 2 blue, Farm 3 green, Farm 4 yellow, and Farm 5 pink.

Phylogenetic analysis of NA gene sequences revealed the circulation of N1 classical swine in prewean and nursery production stages in Farm 3. In addition, N2 2002B sequences were detected in Farm 4 and Farm 5 in both production stages (Figure 7).

The H3 HA nucleotide homology and antigenic motif represented by amino acid positions 145, 155, 156, 158, 159, and 189 from Farm 5 were analyzed and compared to the vaccine sequence provided by the participating farm. For the H3 1990.4 clade vaccine sequence used in the autogenous vaccine, a subsequent farm sequence for H3 1990.4.a showed an 89.71% nucleotide homology with the vaccine HA sequence, while the farm sequence for H3.2010.1 showed an 86.07% HA nucleotide homology (Table 6).

The nursery farm H3 1990.4.a sequence showed a difference at two positions, 156 (K to R) and 158 (N to G), in the antigenic motif compared to the H3 1990.4 vaccine amino acid antigenic motif. The nursery farm H3 2010.1 sequence also indicated a difference in all six positions in the antigenic motif compared to the vaccine strain (Table 6).

## 4. Discussion

This study implemented an active surveillance approach to IAV detection and genetic diversity over time at breeding and nursery farms at the human–swine interface in both pigs and farm employees. Among the surveyed farm employees, only 29.2% reported receiving the IAV vaccine within the past 12 months (Table 2). This rate of vaccination among farm workers was found to be lower than the reported national average (46.9%) of IAV vaccination coverage for the 2022–2023 influenza season in the US, as reported by the Centers for Disease Control and Prevention [27]. In a prior study, 5% of swine workers reported they received the seasonal IAV human vaccine in one year, and 7% received the IAV human vaccine in a consecutive year, highlighting a low percentage of vaccinated farm employees across swine farms [28]. In the current study, the low rate of farm employee vaccination highlights a potential risk of infection and bidirectional transmission of IAV between the workers and swine on the farms, considering repeated evidence of human-seasonal IAV transmission to swine [29,30].

During the active surveillance conducted in this study, where farm employees were asked to self-collect respiratory samples once a month, we demonstrated comparable IAV RNA detection to a study in 2023 that detected IAV RNA using RT-rtPCR in 0.9% (2/229) of employees when sampled at their place of work [31]. In contrast, a recent study in US Midwestern swine farms that assessed IAV detection in farm employees before and after work on the farm detected IAV in 56.9% (33/58) of farm employees [12]. A possible reason for the higher IAV detection in this study may be the farm employee sampling scheme that occurred twice a week for eight consecutive weeks only after the CDC reported an upward trend in influenza-like illnesses (ILIs) during the human influenza season. This may have increased the opportunity of IAV detection in their farm employees. In our study, the sampling occurred randomly only once per month for at least 12 months, regardless of the levels of ILI in the general population. Considering this sampling frequency, some human infections may have been missed, and IAV may have been underrepresented or only detected late during the infection cycle. The proposition that IAV was circulating in some farm employees was supported by our detection and sequencing of two human cases of H3N2, but each of these detections was associated with high RT-rtPCR Ct values.

In this study, three cases of human-seasonal IAV were detected in farm employees while present at work at the farm although no direct evidence of transmission to swine was observed. Nonetheless, human-seasonal IAV spillover to swine occurs somewhat frequently in the USA and globally and has influenced the ecology of IAV in swine for the past century [32]. However, the dynamics that influence IAV transmission at the human–swine interface require further study, as some different factors influence interspecies transmission, e.g., the number of employees and animals in regular contact with each other. This study involved five swine farms and only 17,040 breeding sows, which is lower than one percent of the US sow inventory of approximately 6.01 million breeding sows (https://www.nass.usda.gov/Newsroom/2024/06-27-2024.php, (accessed on 14 August 2024)), suggesting more broad surveillance is needed to increase the detection of human IAV infections that may be occurring in swine farm employees.

Current strains of IAV circulating in swine have originated from human spillover to pigs and established the current H1N1, H1N2, and H3N2 subtypes in swine [10]. The two cases of human-seasonal H3N2 detected in this study (Figure 2) were sequenced by the Sanger method and represent the 2022–2023 human-seasonal IAV from the 3C.2a1b.2a.2b clade. The most recent introductions of human-seasonal influenza H3N2 viruses into swine are represented by H3 2010.1, which originated in 2012, and H3 2010.2, subsequently in 2016 [33]. In this study, the 2022–2023 human-seasonal IAV was not detected in the swine monitored; however, there has been recent evidence of human-seasonal IAV infection in swine that has established another endemic H3 lineage (H3 2020.1) in swine, highlighting the importance of the human–swine interface [34]. Further, when human IAVs spill over, adapt, and become established in swine, they may represent a zoonotic risk with evidence of swine-lineage H3N2v detections that have occurred most often in humans attending agricultural fairs [35].

While all surveyed farms included in this study recommended employee influenza vaccines (Table 4), the low vaccination responses among these farm employees (Table 2) compared to the current IAV human vaccination coverage [27] suggests a potential risk for non-vaccinated farm employees getting infected with either human- or swine-lineage IAV. Only 60% (3/5) of the farms responded that employees use personal protective equipment and 60% (3/5) of the farms recommended implementing a sick leave policy to prevent the spread of IAV among humans and from people to swine. This suggests a gap between biosecurity recommendations and their practical application among different farms, emphasizing the need for consistent and effective biosecurity strategies [36], such as personal protective equipment (PPE), sick leave policies, and routine disinfection to mitigate transmission risks at the human–swine interface [37,38]. Farm employees in swine farms may not only face the risk of IAV exposure, transmission, and infection from swine IAV while working on swine farms [12], but farm workers could also serve as a means for influenza transmission to swine and potentially establishing new endemic IAV in pigs [39]. Therefore, increasing IAV seasonal vaccination coverage among agricultural workers is a critical strategy in the battle against human-to-swine spillover and prevention of zoonotic swine IAV infections [40].

Differences were observed across the various sample types and between the prewean and nursery stages in the farms in IAV detections (Figure 3). Throughout the prewean period, the overall IAV positivity among all sample types was lower compared to the nursery phase, where Ct values were lower than in the prewean phase, suggesting more virus circulating at the time of sample collection. The lower positive rate in prewean pigs is consistent with endemic IAV infections that often occur on breeding farms [41,42], allowing the piglets to play a critical role in maintaining undetected IAV infections [43]. Vaccination of replacement gilts and sows in all farms (Table 3) may decrease transmission and piglet detection in the preweaning stage if vaccine antigens match the circulating strains on the farms. Previous studies have shown that vaccination of sows can reduce IAV transmission among piglets [44], and vaccination of gilts can reduce the prevalence of IAV in breeding herds [45].

Differences in the detection of IAV subtypes in both preweaning piglets and nursery pigs were also observed (Figure 4). Among prewean piglets, RT-rtPCR detected H1N1 and H3N2 subtypes, with two mixed subtype detections in Farm 3. More frequent IAV subtypes in nursery pigs were observed, including H1N1, H3N2, and H1N2 (Figure 4). Mixed IAV PCR subtype detections were identified across four nursery farms, indicating a more diverse array of IAV strains detected within this population, as observed in other studies that showed mixed subtype infections and increased genetic diversity within IAV from nursery pig populations [46,47]. In addition, mixed IAV subtype detections at the same farm present a risk of reassortment that often occurs with IAV, which can influence the genetic diversity of circulating strains at the farm level [48].

While HA and NA gene sequences are sufficient to determine their subtype and genetic clade, generating whole genome sequences of all eight segments provides crucial information regarding their evolution [49]. Influenza A virus is characterized by eight genes, which include the surface glycoproteins HA and NA, and six internal genes, where combinations of these are known as the internal gene constellation [50]. This study consistently observed the internal gene constellation TTTPPT during the prewean and nursery phases in the clades identified in Farms 2, 3, and 4 when whole genomes were generated (Figure 5). The TTTPPT internal gene constellation represents approximately 54% of all detections in US swine between 2020 and 2022 [51], and there is evidence that the internal gene constellation may affect virus transmission efficiency [50]. Farm 1 showed evidence of two different NP circulating between TRIG or PDM lineages. Moreover, in Farm 5, the internal gene PA had evidence of TRIG or PDM lineages circulating with a mixed LAIV and TRIG lineage detected in the PB2 (Figure 5). Using WGS enables the detection and characterization of internal genes, their lineages, and combinations with HA and NA [42], demonstrating the risk of viral reassortment and evolution at the farm level [52] and the risk of the emergence of new IAV [53].

Based on the phylogenetic analysis, Farms 3, 4, and 5 displayed similar IAV sequences in both the prewean and nursery stages, suggesting a role of piglets in spreading IAV to downstream nurseries and potentially to other farms after weaning [54,55]. Specifically, Farms 3 and 5 showed multiple NA detections from oral fluid samples in the nursery phase, indicating viral dynamics and challenges associated with mitigating IAV at the farm level when multiple subtypes or strains are co-circulating on the farm. Farm 4 had H3 1990.4.a and N2 2002B that were consistently detected within this farm, suggesting the presence of only one strain of IAV at the nursery. In contrast, the nursery locations for Farm 5 had five different HA clades detected over time (Figure 5 and Figure 6). The results of the phylogenetic analysis of the HA, NA, and internal gene segments suggest that farm-level IAV genetic diversity may vary or remain constant, depending on multiple factors often unique to the farm, in addition to demonstrating the potential for reassortment among strains when more than one IAV is present on the farm, which ultimately complicates control strategies. Unlike IAV national-level surveillance, which often provides broad, aggregated data, farm-level monitoring offers more granular insights into IAV diversity and dynamics within specific production environments, which is necessary for optimal virus control [56]. Interestingly, the farm survey (Table 6) reported that most nursery farms do not monitor IAV except when the animals present clinical signs. Therefore, implementing comprehensive farm-level surveillance that includes nursery pigs is essential for understanding IAV virus diversity over time. Moreover, it can identify unique IAV strains and patterns that might be missed when surveillance is exclusive to breeding farms and prewean pigs, thus providing crucial data for more effective mitigation strategies tailored to specific farm conditions.

IAV surveillance at the farm level is necessary to understand the diversity and how the IAV clades are evolving, or to detect the introduction of a new IAV clade. In addition, it is also helpful to investigate if an antigenic change in the HA protein is challenging vaccine efficacy. The H3 antigenic motif, comprising amino acid positions 145, 155, 156, 158, 159, and 189, can be used for evaluating antigenic distance and guiding IAV vaccine antigen selection, or to determine when vaccine antigens need updating [57]. Using Farm 5 as an example, this study observed a difference in the antigenic motif between the H3 HA used in the vaccine and two HA sequences generated during the study (Table 6). The vaccine sequence was based upon H3.1990.4, which is a different clade than what was detected in this study, where H3.1990.4.a had two antigenic sites diverging at the 156 and 158 positions compared to the vaccine sequence. In addition, H3.2010.1 was also detected in Farm 5, which is a significantly different lineage from vaccine H3.1990.4 and all its antigenic sites were different compared to the vaccine sequence. This observation suggests that the current vaccine antigen, H3.1990.4, is unlikely to induce cross-protective antibodies to the current circulating strains and requires a vaccine antigen update with relevant strains circulating at the farm [57,58]. Therefore, this field example demonstrates that IAV surveillance with HA sequencing can also inform antigenic differences between vaccine and farm strains, and when vaccine antigens should be updated to maintain optimal control at the farm level.

This study has some limitations that should be considered when interpreting the results. The farm employee samples were self-collected, and in some months the farm employees did not collect the sample due to unknown factors, and the sampling frequency for PCR surveillance in farm employees was inconsistent. Moreover, the number of samples collected may not detect the presence of IAV in a swine population in the scenario of extremely low prevalence, and the selection of animals for sample collection was not based on clinical signs as this was not the objective, considering the study was performed using a predefined, active surveillance approach on the swine farms. In addition, swine samples were not collected and submitted in some months. In this case, the inconsistency in sampling frequency could result in certain months or individuals being overrepresented or underrepresented, leading to a selection bias [59]. This study was conducted in Midwestern US swine farms, and these results might be different in other regions as IAV circulation is dynamic and changes over time.

## 5. Conclusions

This study provided comprehensive insights into IAV detection and diversity within swine farms and among farm employees at the human–swine interface. Notably, the presence of H3N2 in farm employees from the 2022–2023 human IAV season demonstrated that farm employees could harbor human-seasonal IAV in their respiratory tract while working closely with swine, highlighting the importance of biosecurity measures at the human–swine interface. This also highlights that while continuous surveillance is essential, addressing the risk of IAV transmission from humans to swine is equally important. This study emphasizes the need for ongoing surveillance and robust biosecurity interventions to monitor IAV dynamics within swine farms and mitigate the potential introduction and spread of human-derived IAV in swine populations.

## Figures and Tables

**Table 1:** Sampling protocol for farm employees and swine for active surveillance by each farm.

Category	Sample Type	Number per Month	Age Target	Total Samples Collected
Farm employee	Nasal secretion	≥2	≥18 years old	447
Prewean piglets	Udder wipes	10	2–3 weeks-old	486
Prewean piglets	Nasal wipes	15	2–3 weeks-old	712
Nursery pigs	Nasal wipes	15	6–9 weeks-old	742
Nursery pigs	Oral fluids	4	6–9 weeks-old	218

**Table 2:** Overview of farm employee demographics at enrollment.

Variable	Category	Frequency (n)	Percent (%)
Farm employee age (years)	18–25	8	16.7
	26–35	14	29.2
	36–45	15	31.3
	46–55	10	20.7
	>56	6	2.1
Farm employee gender	Male	30	62.5
	Female	18	37.5
Farm employee influenza vaccination (Previous 12 months)	Yes	14	29.2
	No	32	66.7
	Do not know	2	4.1
Time working with swine (years)	1 year	13	27.1
	1–2 years	5	10.4
	2–5 years	8	16.7
	5–10 years	8	16.7
	>10 years	14	29.1

**Table 3:** Breeding herd demographics representing the prewean piglet population.

Variable	Category	Number of Farms	Percent (%)
Average sow inventory	1000–5000	3	60
	>5000	2	40
Farrowing system	Batch flow	1	20
	Continuous flow	4	80
Frequency of weaning piglets	Weekly	4	80
	Every three weeks	1	20
Frequency of gilts entering the breeding herd per year	Weekly	2	40
	Monthly	2	40
	Quarterly (every 3 months)	1	20
Internal gilt multiplication	Yes	2	40
	No	3	60
Gilt influenza vaccination	Yes	5	100
	No	0	0
Influenza vaccine doses prior to breeding herd entry	1 dose	0	0
	2 doses	4	80
	3 doses	1	20
Vaccine administration: weeks prior to breeding herd entry	1 week	0	0
	2–3 weeks	1	20
	>3 weeks	4	80
Whole-herd influenza vaccination	Yes	3	60
	No	2	40
Influenza vaccination frequency	Once per year	0	0
	Twice or more per year	3	60
	Pre-farrow administration	1	20
	Other	1	20
Influenza vaccine product	Autogenous or farm-specific	5	100
	Commercial vaccine	0	0
Number of vaccine antigens	4 strains per dose	1	20
	5 strains per dose	4	80

**Table 4:** Breeding herd influenza A virus diagnostic testing and surveillance, biosecurity, and biomanagement strategies.

Variable	Category	Frequency	Percent (%)
Diagnostic testing and surveillance			
Routine influenza surveillance	Yes	3	60
	No	2	40
Gilt source influenza virus status	Gilts influenza positive	1	20
	Gilts influenza negative	3	60
	Influenza status unknown	1	20
Gilt source influenza antibody status	Influenza antibody positive	1	20
	Influenza antibody negative	1	20
	Influenza antibody unknown	3	60
The goal of influenza surveillance	Targeting IAV control	1	20
	Targeting IAV elimination	1	20
	No specific IAV protocol	1	20
	Unknown	2	40
Biosecurity			
Employee uses personal protective equipment	Yes	3	60
	No	2	40
Farm employees are influenza-vaccinated	Yes	4	80
	No	1	20
Farm recommends employee influenza vaccine	Yes	5	100
	No	0	0
Farm recommends use of sick leave policy	Yes	3	60
	No	2	40
Biomanagement			
The farm uses nurse sows	Yes	5	100
	No	0	0
Nurse sows moved between farrowing rooms	Yes	4	80
	No	1	20

**Table 5:** Nursery pig influenza A virus diagnostic testing and surveillance during the study.

Variable	Category	Frequency	Total (%)
Pig source at the nursery site	Single source	3	60
	Mixed source	2	40
Influenza surveillance conducted at the nursery	Yes	1	20
	No	4	80
Nursery pigs receive IAV vaccine	Yes	0	0
	No	5	100
Diagnostics conducted for influenza in nursery	Yes	1	20
	No	4	80
Number of influenza vaccine doses	1 dose	0	0
	2 doses	0	0
	No vaccination	5	100

**Table 6:** Farm 5 H3 hemagglutinin vaccine sequence and H3 hemagglutinin nursery phase sequence nucleotide homology and antigenic motif.

Farm	Sequence Source	H3 Clade	Date	Nucleotide Homology *	Antigenic Motif **					
					145	155	156	158	159	189
5	Vaccine sequence	H3.1990.4	October 2021	-	G	C	K	N	S	K
5	Nursery Sequence	H3.1990.4.a	May 2023	89.71%	G	C	R	G	S	K
5	Nursery Sequence	H3.2010.1	July 2023	86.07%	D	V	R	G	M	I

* H3 nucleotide homology based on comparing vaccine and nursery farm H3 HA sequences. ** Antigenic motif amino acids in bold indicate differences between vaccine and nursery farm H3.

## Data Availability

The data presented in this study are available upon request from the corresponding authors.

## Supplementary Materials

The following supporting information can be downloaded at: https://www.mdpi.com/article/10.3390/v16121921/s1, Supplementary Material A: Farm Questionnaire; Table S1: Influenza A virus detected in five swine farms including the phylogenetic tree taxa, influenza A virus designation, and HA or NA GenBank accession number; Table S2: Farm survey responses regarding Influenza A virus management practices reported lower and higher IAV positivity categories.
