# Supplementary material for: Influenza A Virus Detection at the Human–Swine Interface in US Midwest Swine Farms
Source: Viruses. 2024 Dec 15;16(12):1921. doi: 10.3390/v16121921 (PMC11680338; doi:10.3390/v16121921)
Supplement: Supplementary file 1 [file viruses-16-01921-s001.zip › Supplemental material A.pdf]

Supplemental material A
Farm Questionnaire

**Date:**	
**Farm name:**	
**Veterinarian:**	

PART 1: SOW FARM:

1. Herd size: Average sow inventory: Describe:
    a. Number of replacement gilts entering the breeding herd per year: Describe:
    b. What is your gilt schedule frequency to enter the breeding herd (i.e., weekly, monthly, quarterly, yearly) Describe:
2. Farrowing system:
    a. Batch flow
    b. Continuous flow
3. Frequency of weaning piglets
    a. Weekly
    b. Every three weeks
4. Frequency of gilts entering the breeding herd per year
    a. Weekly
    b. Monthly
    c. Quarterly (every 3 months)
5. Internal gilt multiplication
    a. Yes
    b. No
6. Gilt influenza vaccination
    a. Yes
    b. No
7. Influenza vaccine doses prior to breeding herd entry
    a. 1 dose

    b. 2 doses
    c. 3 doses

8. Vaccine administration: weeks prior to breeding herd entry
    a. <1 week
    b. 2-3 weeks
    c. >3 weeks
9. Whole-herd influenza vaccination
    a. Yes
    b. No
10. Influenza vaccination frequency
    a. Once per year
    b. Twice or more per year
    c. Pre-farrow administration
    d. Other
11. Influenza vaccine product
    a. Autogenous or farm-specific
    b. Autogenous or farm-specific
12. Number of vaccine antigens
    a. 4 strains per dose
    b. 5 strains per dose
13. Routine influenza surveillance
    a. Yes
    b. No
14. Gilt source influenza virus status
    a. Gilts influenza positive
    b. Gilts influenza negative
    c. Influenza status unknown

15. Gilt source influenza anti-body status
    a. Influenza antibody positive

    b. Influenza antibody negative
    c. Influenza antibody unknown

16. The goal of influenza surveillance
    a. Targeting IAV control
    b. Targeting IAV elimination
    c. No specific IAV protocol
    d. Unknown
17. Employee uses personal protective equipment
    a. Yes
    b. No
18. Farm employees are influenza-vaccinated
    a. Yes
    b. No
19. Farm recommends employee influenza vaccine
    a. Yes
    b. No
20. Farm recommends use of sick leave policy
    a. Yes
    b. No
21. The farm uses nurse sows
    a. Yes
    b. No
22. Nurse sows moved between farrowing rooms
    a. Yes
    b. No
23. Pig source at the nursery site
    a. Single source
    b. Mixed source
24. Influenza surveillance conducted at the nursery
    a. Yes

    b. No

25. Nursery pigs receive IAV vaccine
    a. Yes
    b. No
26. Diagnostics conducted for influenza in nursery
    a. Yes
    b. No
27. Number of influenza vaccine doses
    a. 1 dose
    b. 2 doses
    c. No vaccination
