# Supplementary material for: Influenza A Virus Detection at the Human–Swine Interface in US Midwest Swine Farms
Source: Viruses. 2024 Dec 15;16(12):1921. doi: 10.3390/v16121921 (PMC11680338; doi:10.3390/v16121921)
Supplement: Supplementary file 1 [file viruses-16-01921-s001.zip › Supplemental material Table S2.pdf]

Supplemental material

Table S2. Farms survey responses regarding Influenza A virus management practices reported lower and higher IAV positivity categories.

Variable	Category	Positivity Category		Total %	*P-value*
		Lower Positivity %	Higher Positivity %		
**Breeding herds**					
Average sow inventory	1000-5000	60 (3/5)	0 (0/5)	60 (3/5)	0.4
	>5000	20 (1/5)	20 (1/5)	40 (2/5)	
Farrowing system	Batch flow	20 (1/5)	0 (0/5)	20 (1/5)	0.99
	Continuous flow	60 (3/5)	20 (1/5)	80 (4/5)	
Frequency of weaning piglets	Weekly	60 (3/5)	20 (1/5)	80 (4/5)	0.99
	Every three weeks	20 (1/5)	0 (0/5)	20 (1/5)	
Frequency of gilts entering the breeding herd per year	Weekly	40 (2/5)	0 (0/5)	40 (2/5)	0.99
	Monthly	20 (1/5)	20 (1/5)	40 (2/5)	
	Quarterly	20 (1/5)	0 (0/5)	20 (1/5)	
Internal gilt multiplication	Yes	40 (2/5)	0 (0/5)	40 (2/5)	0.99
	No	40 (2/5)	20 (1/5)	60 (3/5)	
Gilt influenza vaccination	Yes	80 (4/5)	20 (1/5)	100 (5/5)	0.99
	No	0 (0/5)	0 (0/5)	0 (0/5)	
Influenza vaccine doses prior to breeding herd entry	1 dose	20 (1/5)	0 (0/5)	20 (1/5)	0.99
	2 doses	40 (2/5)	20 (1/5)	60 (3/5)	
	3 doses	20 (1/5)	0 (0/5)	20 (1/5)	
Vaccine administration: weeks prior to breeding herd entry	1 week	20 (1/5)	0 (0/5)	20 (1/5)	0.99
	2-3 weeks	20 (1/5)	0 (0/5)	20 (1/5)	
	> 3 weeks	40 (2/5)	20 (1/5)	60 (3/5)	
Whole-herd influenza vaccination	Yes	60 (3/5)	0 (0/5)	60 (3/5)	0.4
	No	20 (1/5)	20 (1/5)	40 (2/5)	
Influenza vaccination frequency	Once per year	0 (0/5)	0 (0/5)	0 (0/5)	0.4
	Twice or more per year	60 (3/5)	0 (0/5)	60 (3/5)	
	Pre-farrow administration	0 (0/5)	20 (1/5)	20 (1/5)	
	Other	20 (1/5)	0 (0/5)	20 (1/5)	
Influenza vaccine product	Autogenous	80 (4/5)	20 (1/5)	100 (5/5)	0.99
	Commercial vaccine	0 (0/5)	0 (0/5)	0 (0/5)	
Number of vaccine antigens	4 strains per dose	0 (0/5)	20 (1/5)	20 (1/5)	0.2
	5 strains per dose	80 (4/5)	0 (0/5)	80 (4/5)	
**Diagnostic testing and surveillance**					
	Yes	60 (3/5)	0 (0/5)	60 (3/5)	0.4

Routine influenza surveillance at the breeding herds	No	20 (1/5)	20 (1/5)	40 (2/5)	
Gilt source influenza virus status	Gilts influenza positive	20 (1/5)	0 (0/5)	20 (1/5)	0.4
	Gilts influenza negative	60 (3/5)	0 (0/5)	60 (3/5)	
	Influenza status unknown	0 (0/5)	20 (1/5)	20 (1/5)	
Gilt source influenza antibody status	IAV antibody positive	20 (1/5)	0 (0/5)	20 (1/5)	0.99
	IAV antibody negative	0 (0/5)	0 (0/5)	0 (0/5)	
	IAV antibody unknown	60 (3/5)	20 (1/5)	80 (4/5)	
The goal of influenza surveillance	Targeting IAV control	20 (1/5)	0 (0/5)	20 (1/5)	0.6
	Targeting IAV elimination	0 (0/5)	20 (1/5)	20 (1/5)	
	No specific IAV protocol	20 (1/5)	0 (0/5)	20 (1/5)	
	Unknown	40 (2/5)	0 (0/5)	40 (2/5)	
**Biosecurity**					
Employee uses personal protective equipment	Yes	60 (3/5)	0 (0/5)	60 (3/5)	0.4
	No	20 (1/5)	20 (1/5)	40 (2/5)	
Farm employees are influenza-vaccinated	Yes	60 (3/5)	20 (1/5)	80 (4/5)	0.99
	No	20 (1/5)	0 (0/5)	20 (1/5)	
Farm recommends employee influenza vaccine	Yes	80 (4/5)	20 (1/5)	100 (5/5)	0.99
	No	0 (0/5)	0 (0/5)	0 (0/5)	
Farm recommends use of sick leave policy	Yes	60 (3/5)	0 (0/5)	60 (3/5)	0.4
	No	20 (1/5)	20 (1/5)	40 (2/5)	
**Biomanagement**					
The farm uses nurse sows	Yes	80 (4/5)	20 (1/5)	100 (5/5)	0.99
	No	0 (0/5)	0 (0/5)	0 (0/5)	
Nurse sows moved between farrowing rooms	Yes	60 (3/5)	20 (1/5)	80 (4/5)	0.99
	No	20 (1/5)	0 (0/5)	20 (1/5)	
**Nursery**					
Pig source at the nursery site	Single	20 (1/5)	40 (2/5)	60 (3/5)	0.99
	Mixed	0 (0/5)	40 (2/5)	40 (2/5)	
Routine influenza surveillance at the breeding herds at the nursery	Yes	20 (1/5)	0 (0/5)	20 (1/5)	0.2
	No	0 (0/5)	80 (4/5)	80 (4/5)	
Nursery pigs receive IAV vaccine	Yes	0 (0/5)	0 (0/5)	0 (0/5)	0.99
	No	20 (1/5)	80 (4/5)	100 (5/5)	
	Yes	20 (1/5)	0 (0/5)	20 (1/5)	0.2

Diagnostics conducted for influenza in nursery	No	0 (0/5)	80 (4/5)	80 (4/5)	
Number of influenza vaccine doses	1 dose	0 (0/5)	0 (0/5)	0 (0/5)	0.99
	2 doses	0 (0/5)	0 (0/5)	0 (0/5)	
	No vaccination	20 (1/5)	80 (4/5)	100 (5/5)	
